# Supplementary material for: Heat adaptation of phage T7 under an extended genetic code
Source: Virus Evol. 2021 Dec 1;7(2):veab100. doi: 10.1093/ve/veab100 (PMC8923235; doi:10.1093/ve/veab100)
Supplement: veab100_Supp [file veab100_supp.zip › Sequencing_Analysis.zip!/Sequencing_Analysis/A3/evidence/JC_SIDE_1_52.html]

BRESEQ :: Evidence


| New junction evidence | | | | | | | | | | | |
| --- | --- | --- | --- | --- | --- | --- | --- | --- | --- | --- | --- |
|  | | seq id | position | reads (cov) | reads (cov) | score | skew | freq | annotation | gene | product |
| \* | ? | NC\_001604.1 | = 281 | 1741 (1.120) | 373 (0.220) +TAAAGACCAGACC | 41/508 | 22.1 | 16.7% | intergenic (–/–) | *–/–* | –/– |
|  | ? | NC\_001604.1 | 282 = | 1933 (1.110) |  |  |  |  | intergenic (–/–) | *–/–* | –/– |
| Rejected: Coverage evenness skew score above cutoff. | | | | | | | | | | | |
| Rejected: Frequency below/above cutoff threshold. | | | | | | | | | | | |

Only 100 of 2303 total aligned reads displayed.  

`‑‑‑‑‑‑‑‑‑‑‑TCTCACAGTGTACGGACCTAAAGTTCCCCCATAGGGGGTACCTAAAGCCCAGCCAATCACCTAAAGTCAACCTTCGGTTGACCTTGAGGGTTCCCTAAGGGTTGGGGATGACCCTTGGGTTTGTCTTTGGGTGTTACCTTGAGTGTCTCTCTGTGT‑CCCTATCTGTTACAGTCTCCTAAAGTATCCTCCTAAAGTCACCTCCTAACGTCCATCCTAAAGCCAACACCTAAAGCCTACACCTAAAGACCCATCAAGTCAACGCCTATCTTAAAGTTTAAACATAAAGACCAGACCTAAAGACCAGACCTAAAGACACTACATAAAGACCAGACCTAAAGACGCCTTGTTGTTAGCCATAAAGTGATAACCTTTAATCATTGTCTTTATTAATACAACTCACTATAAGGAGAGACAACTTAAAGAGACTTAAAAGATTAATTTAAAATTTATCAAAAAGAGTATTGACTTAAAGTCTAACCTATAGGATACTTACAGCCATCGAGAGGGACACGGCGAATAGCCATCCCAATCGACACCGGGGTCAACCGGATAAGTAGACAGCCTGATAAGTCGCACGAAAA  >  NC_001604.1/1‑581`  

                                                                                                                                                                                                                                                                                                    `|`                                                                                                                                                                                                                                                                                                              
`gatgtgtataagagacaggtgtACGGACCTAAAGTTCCCCCATAGGGGGTACCTAAAGCCCAGCAAATCACCTAAAGTCAACCTTCGGTTGACCTTGAGGGTTCCCTAAGGGTTGGGGATGACCCTTGGGTTTGTCTTTGGGTGTTACCTTGAGTGTCTCTCTGTGTCCCCTATCTGTTACAGTCTCCTAAAGTATCCTCCTAAAGTCACCTCCTAACGTCCATCCTAAAGCCAACACCTAAAGCCTACACCTAAAGACCCATCAAGTCAACGCCTATCTTAAAGTTTAAACAtaaagacc                                                                                                                                                                                                                                                                                                      <  1:61029/283‑1 (MQ=255)`  
  `tgtgtataagagacaggtgtACGGACCTAAAGTTCCCCCATA‑GGGGTACCTAAAGCCCAGCCAATCACCTAAAGTCAACCTTCGGTTGACCTTGAGGGTTCCCTAAGGGTTGGGGATGACCCTTGGGTTTGTCTTTGGGTGTTACCTTGAGTGTCTCTCTGTGT‑CCC‑ATCTGTTACAGTCTCCTAAAGTATCCTCCTAAAGTCACCTCCTAACGTCCATCCTAAAGCCAACACCTAAAGCCTACACCTAAAGACCCATCAAGTCAACGCCTATCTTAAAGTTTAAACAtaaagaccagacc                                                                                                                                                                                                                                                                                                 <  1:258072/285‑1 (MQ=255)`  
   `gtgtataagagacaggtgtACGGACCTAAAGTTCCCCCATAGGGGGTACCTAAAGCCCAGCCAATCACCTAAAGTCAACCTTCGGTTGACCTTGAGGGTTCCCTAAGGGTTGGGGATGACCCTTGGGTTTGTCTTTGGGTGTTACCTTGAGTGTCTCTCTGTGT‑CCCTATCTGTAACAGTCTCCTAAAGTATCCTCCTAAAGTCACCTCCTAACGTCCATCCTAAAGCCAACACCTAAAGCCTACACCTAAAGACCCATCAAGTCAACGCCTATCTTAAAGTTTAAACAtaaagaccagac                                                                                                                                                                                                                                                                                                  <  1:214965/286‑1 (MQ=255)`  
            `agcttcaggtgcGGACCTAACGTTCCCCCATAGGGGGTACCTACAGCCCAGCCAATCACTTAAAGTCAACCTTCGGTTGACCTTGAGGGTTCCCTAAGGGTTGGGGATGACCCTTGGGTTTGTCTTTGGGTGTTACCTTGAGTGTCTCTCTGTGTCCCCTATCTGTTACAGTCTCCTAAAGTATCCTCCTAAAGTCACCTCCTAACGTCCATCCTAAAGCCAACACCTAAAGCCTACACCTAAAGACCCATCAAGTCAACGCCTATCTTAAAGTTTAAACAtaaagaccagacctaaagac                                                                                                                                                                                                                                                                                          <  1:300101/290‑1 (MQ=255)`  
                  `gtgtaccacccacaaGTTCCCCCATAGGGGGTACCTAAAGCCCAGCCAATCACCTAAAGTCAACCTTCGGTTGACCTTGAGGGTTCCCTAAGGGTTGGGGATGACCCTTGGGTTTGTCTTTGGGTGTTACCTTGAGTGTCTCTCTGTGT‑CCCTATCTGTAACAGTCTCCTAAAGTATCCTCCTAAAGTCACCTCCTAACGTCCATCCTAAAGCCAACACCTAAAGCCTACACCTAAAGACCCATCAAGTCAACGCCTATCTTAAAGTTTAAACAtaaagaccagaccctgtctcttataca                                                                                                                                                                                                                                                                                   >  1:322330/14‑287 (MQ=255)`  
                  `gtgtACGTACCTAAAGTTCCCCCATAGGGGGTACCTAAAGCCCAGCCAATCACCTAAAGTCAACCTTCGGTTGACCTTGAGGGTTCCCTAAGGGTTGGGGATGACCCTTGGGTTTGTCTTTGGGTGTTACCTTGAGTGTCTCTCTGTGT‑CCCTATCTGTAACAGTCTCCTAAAGTATCCTCCTAAAGTCACCTCCTAACGTCCATCCTAAAGCCAACACCTAAAGCCTACACCTAAAGACCCATCAAGTCAACGCCTATCTTAAAGTTTAAACATaaagaccagacctaaagaccagacct                                                                                                                                                                                                                                                                                   >  1:112280/1‑301 (MQ=255)`  
                  `gtgtACGGACCTAAAGTTCCCCCATAGGGGGTACCTAAAGCCCAGCCAATCACCTAAAGTCAACCTTCGGTTGACCTTGAGGGTTCCCTAAGGGTTGGGGATGACCCTTGGGTTTGTCTTTGGGTGTTACCTTGAGTGTCTCTCTGTGTCCCCTATCTGTTACAGTCTCCTAAAGTATCCTCCTAAAGTCACCTCCTAACGTCCATCCTAAAGCCAACACCTAAAGCCTACACCTAAAGACCCATCAATTCAACGCCTACCTTAAAGTTTAAACAtaaagacaagacctaaagacaaaccc                                                                                                                                                                                                                                                                                    >  1:1867/1‑295 (MQ=255)`  
                  `gtgtACGGACCTAAAGTTCCCCCATAGGGGGTACCTAAAGCCCAGCCAATCACCTAAAGTCAACCTTCGGTTGACCTTGAGGGTTCCCTAAGGGTTGGGGATGACCCTTGGGTTTGTCTTTGGGTGTTACCTTGAGTGTCTCTCTGTGTCCCCTATCTGTTACAGTCTCCTAAAGTATCATCCTAAAGTAACCTCCTAACGTCCATCCTAAAGCCAACACCTAAAGCCTACACCTAAAGACCCATCAAGTCAACGCCTATCTTAAAGTTTAAACAtaaagaccagacctaaagaccagacc                                                                                                                                                                                                                                                                                    >  1:103523/1‑301 (MQ=255)`  
                  `gtgtACGGACCTAAAGTTCCCCCATAGGGGGTACCTAAAGCACAGCCAATCACCTAAAGTCAACCTTCGGTTGACCTTGAGGGTTCCCTAAGGGTTGGGGATGACCCTTGGGTTTGTCTTTGGGTGTTACCTTGAGTGTCTCTCTGTGT‑CCC‑ATCTGTTACAGTCTCCTAAAGTATCCTCCTAAAGTCACCTCCTAACGTCCATCCTAAAGCCAACACCTAAAGCCTACACCTAAAGACCCATCAAGTCAACGCCTATCTTAAAGTTTAAACATAaaaaccagacctaaagaccagaccta                                                                                                                                                                                                                                                                                  >  1:6374/1‑301 (MQ=255)`  
                    `gtACGGACCTAAAGTTCCCCCATAGGGGGTACCTAAAGCCCAGCCAATCACCTAAAGTCAACCTTTGGTTGACCTTGAGGGTTCCCTAAGGGTTGGGGATGACCCTTGGGTTTGTCTTTGGGTGTTACCTTGAGTGTCTCTCTGTGT‑CCC‑ATCTGTTACATTCTCCTAAAGTATCCTCCTAAAGTCACCTCCTAACGTCCATCCTAAAGCCAACACCTAAAGCCTACACCTAAAGACCCATCAAGTCAACGCCTATCTTAAAGTTTAAACATAAAgaccagacctaaagaccagacctaaa                                                                                                                                                                                                                                                                                >  1:246280/1‑301 (MQ=255)`  
                    `gtACGGACCTAAAGTTCCCCCATAGGGGGTACCTAAAGCCCAGCCAATCACCTAAAGTCAACCTTCGGTTGACCTTGAGGGTTCCCTAAGGGTTGGGGATGACCCTTGGGTTTGTCTTTGGGTGTTACCTTGAGTGTCTCTCTGTGTCCCCTATCTGTAACAGTCTCCTAAAGTATCCTCCTAAAGTCACCTCCTAACGTCCATCCTAAAGCCAACACCTAAAGCCTACACCTAAAGACCAATCAAGTCAACGCCTATCTTAAAGTTTAAACATAaagacaagacctaaagaccagaccta                                                                                                                                                                                                                                                                                  >  1:39837/1‑301 (MQ=255)`  
                    `gtACGGACCTAAAGTTCCACCATAGGGGGTACCTAAAGCCCAGCCAATCACCTAAAGTCAACCTTCGGTTGACCTTGAGGGTTCCCTAAGGGTTGGGGATGACCCTTGGGTTTGTCTTTGGGTGTTACCTTGAGTGTCTCTCTGTGTCCCCTATCTGTTACAGTCTCCTAAAGTATCCTCCTAAAGTCACCTCCTAACGTCCATCCTAAAGCCAACACCTAAAGCCTACACCTAAAGACCCATCAAGTCAACGCCTATCTTAAAGTTTAAACAtaaagacaagacctaaagacctcgtctt                                                                                                                                                                                                                                                                                  >  1:126965/1‑294 (MQ=255)`  
                         `ggactgtaggtgccccATAGGGGGTACCTAAAGCCCAGCCAATCACCTAAAGTCAACCTTCGGTTGACCTTGAGGGTTCCCTAAGGGTTGGGGATGACCCTTGGGTTTGTCTTTGGGTGTTACCTTGAGGGTCTCTCTGTGTCCCCTATCTGTTACAGTCGCCTAAAGTATCCGCCTAAAGTCACATCCTAACGTCCATCCTAAAGCCAACACCTAAAGCCTACACCAAAAGACCCATCAAGTCAACGCCAATCTTAAAGTTTAAACATAAGGACcagacctaaagaccagacctaaagac                                                                                                                                                                                                                                                                             <  1:318281/289‑1 (MQ=25)`  
                         `gACCTAAAGTTCCCCCATAGGGGGTACCTAAAGCCCAGCCAATCACCTAAAGTCAACCTTCGGTTGACCTTGAGGGTTCCCTAAGGGTTGGGGATGACCCTTGGTTTTGTCTTTGGGTGTTACCTTGAGTGTCTCTCTGTGT‑CCCTATCTGTAACATTCTCCTAAAGTATCCTCCTAAAGTCACCTACTAACGTCCATCCTAAAGCCAACACCTAAAGCCTACACCTAAAGCCCCATCAAGTCAACGCCTATCTTAAAGTTTAAACATAAAGACcagacctaaagaccagacctaaagacc                                                                                                                                                                                                                                                                            >  1:165173/1‑300 (MQ=255)`  
                         `gACCTAAAGTTCCCCCATAGGGGGTACCTAAAGCCCAGCCAATCACCTAAAGTCAACCTTCGGTTGACCTTGAGGGTTCCCTAAGGGTTGGGGATGACCCTTGGGTTTGTCTTTGGGTGTTACCTTGAGTGTCTCTCTGTGT‑CCCTATCTGTAACAGTCTCCTAAAGTATCCTCCTAAAGTCACCTCCTAACGTCCATCCTAAAGCCAACACCTAAAGCCTACACCTAAAGACCCATCAAGTCAACGCCTATCTTAAAGTTTAAACATAAAGaccagacctaaagaacagacctaaagctg                                                                                                                                                                                                                                                                            >  1:177657/1‑298 (MQ=255)`  
                         `gACCTAAAGATCCCCCATAGGGTGTACCTAAAGCCCAGCCAATCACCTAAAGTCAACCTTCGGTTGACCTTGAGGGTTCCCTAAGGGTTGGGGATGACCCTTGGGTTTGTCTTTGGGTGTTACCTTGAGTGTCTCTCTGTGT‑CCCTATCTGTAACAGTATCCTAAAGTATCCTCCTAAAGTCACCTCCTACCGTCCATCCTAAAGCCACCACCTAAAGCCTACACCTAAAGACCAATCAAGTCAACGCCTATCTTAAAGTTTAAACATAAagaccagacataaagaccagacctaacgctg                                                                                                                                                                                                                                                                            >  1:292249/1‑296 (MQ=255)`  
                                   `aagagacaggggggTACCTAAAGCCCAGCCAATCACCTAAAGTCAACCTTCGGTTGCCCTTGAGGGTTCCCTAAGGGTTGGGGATGACCCTTGGGTTTGTCTTTGGGTGTTACCTTGAGTGTCTCTCTGTGT‑CCCTAGCTGTAACAGTCTCCTAAAGTATCCTCCTAAAGTCACCTCCTAACGTCCATCCTAAAGCCAACACCTAAAGCCTACACCTAAAGACCCATCAAGTCAACGCCTAACTTAAAGTTTAAACATAAAGACCAGACCTAAAGCCCAGACCTAAAGACACTACATAAag                                                                                                                                                                                                                                                                  <  1:307604/292‑1 (MQ=255)`  
                                      `cccATAGGGGGTACCTAAAGCCCAGCCAATCACCTAAAGTCAACCTTCGGTTGACCTTGAGGGTTCCCTAAGGGTTGGGGATGACCCTTGGGTTTGTCTTTGGGTGTTACCTTGAGTGTCTCTCTGTGT‑CCCTATCTGTAACAGTCTCCTAAAGTATCCTCCTAAAGTCACCTCCTAACGTCCATCCTAAAGCCAACACCTAAAGCCTACACCTAAAGACCCATCAAGTCAACGCCTATCTTAAAGTTTAAACATAAAGACCAGACCTAAAGACCAGACCTAAAGACACTACATAAagctg                                                                                                                                                                                                                                                               >  1:57004/1‑298 (MQ=255)`  
                                             `agacagccTAAAGCCCAGCCAATCACCTAAAGTCAACCTTTGGTTGACCTTGAGGGTTCCCTAAGGGTTGGGGATGACACTTGGGTTTGTCTTTGGGTGTTACCTTGAGTGTCTCTCTGTGA‑CCCTATCTG‑TACAGTCTCCTACAGTATCCTCCTACAGTCACCTACTAACGTCCATCCTAAAGCCAACAACTAAAGCCTACAACTAAAGACCCCTCAAGTAAACGCCTATCTTAAAGTATAAACATAAAGACCAAACCTAACGACCAGACCTCAAGACACTAAATAAagtacacacatca                                                                                                                                                                                                                                                       >  1:239489/7‑290 (MQ=25)`  
                                                 `gaCCTAAAGCCCAGCCAATCACCTAAAGTCAACCTTCGGTTGACCTTGAGGGTTCCCTAAGGGTTGGGTATGACCCTTGGGTTTGTCTTTGGGTGTTACCTTGAGTGTCTCTCTGTGT‑CCCTATCTGTAACAGTCTCCTAAAGTATCCTCCTAAAGTCACCTCCTAACGTCCATCCTAAAGCCAACACCTAAAGCCTACACCTAAAGACCCATCAAGTCAACGCCTATCTTAAAGTTTAAACATAAAGACCAGACCTAAAGACCAGACCTAAAGACACTACATAAAGACCAGACCTAAAGa                                                                                                                                                                                                                                                    <  1:314277/300‑1 (MQ=255)`  
                                                   `ccTAAAGCCCAGCCAATCACCTAAAGTCAACCTTCGGTTGACCTTGAGGGTTCCCTAAGGGTTGGGGATGACCCTTGGGTTTGTCTTTGGGTGTTACCTTGAGTGTCTCTCTGTGT‑CCCTATCTGTAACAGTCTCCTAAAGTATCCTCCTAAAGTCACCTCCTAACGTCCATCCTAAAGCCAACACCTAAAGCCTACACCTAAAGACCCATCAAGTCAACGCCTATCTTAAAGTTTAAACATAAAGACCAGACCTAAAGACCAGACCTAAAGACACTACATAAAGACCAGACCTAAAGAcc                                                                                                                                                                                                                                                  >  1:261951/1‑300 (MQ=255)`  
                                                                              `cAACCGTCGGTTGGCCTTGAGGGTTCCCTAAGGGTTGGGGATGACCCTTGGGTTTGTCTTTGGGTGTTACCTTGAGTGTCTCTCTGTGT‑CCC‑ATCTGTTACAGTCTCCTAAAGTATCCTCCTAAAGTCACCTCCTAACGTCCATCCTAAAGCCAACACCTAAAGCCTACACCTAAAGACCCATCAAGTCAACGCCTATCTTAAAGTTTAAACATAAAGACCAGACCTAAAGACCAGACCTAAAGACACTACATAAAGACCAGACCTAAAGACGCCTTGTTGTTAGCCATAAAGTGATAAcc                                                                                                                                                                                                                      <  1:264432/301‑1 (MQ=255)`  
                                                                                `aCCTTCGGTTGACCTTGAGGGTTCCCTAATGGTTGGGGATGACCCTTGGGTTTGTCTTTGGGTGTTACCTTGAGTGTCTCTCTGTGT‑CCCTATCTGTAACAGTCTCCTAAAGTATCCTCCTAAAGTCACCTCCTAACGTCCATCCTAAAGCCAACACCTAAAGCCTACACCTAAAGACCCATCAAGTCAACGCCTATCTTAAAGTTTAAACATAAAGACCAGACCTAAAGACCAGACCTAAAGAAACTACATAAAGACCAGACCTAAAGACGCCTTGTTGTTAGCCATAAAGTGATAAACt                                                                                                                                                                                                                     >  1:118078/1‑301 (MQ=255)`  
                                                                                `aCCTTCGGTTGACCTTGAGGGTTCCCTAAGGGTTGGGGATGACCCTTGGGTTTGTCTTTGGGTGTTACCTTGAGTGTCTCTCTGTGT‑CCCTATCTGTAACAGTCTCCTAAAGTATCCTCCTAAAGTCACCTCCTAACGTCCATCCTAAAGCCAACACCTACAGCCTACACCTAAAGACCCATCAAGTCAACGCCTATCTTAAAGTTTAAACATAAAGACCAGACCTAAAAACCAGACCTAAAGACACTACATAAAGACCAGACCTAAAGACGCCTTGTTGTTAGCCATAAAGTGATAAccc                                                                                                                                                                                                                     >  1:338266/1‑300 (MQ=255)`  
                                                                                `aCCTTCGGTTGACCTTGAGGGTTCCCTAAGGGTTGGGGATGACCATTGGGTTTGTCTTTGGGTGTTACCTTGAGTGTCTCTCTGTGTCCCCTATCTGTTACAGTCTCCTAAAGTATCCTCCTAAAGTCACCTCCTAACGTCCATCCTAAAGCCAACACCTAAAGCCTACACCTAAAGACCCATCAAGTCAACGCCTATCTTAAAGTTTAAACATAAAGACCAGACCTAAAGACCAGACCTAAAGACACTACATAAAGACCAGACCTAAAGACGCCTTGTTGTTAGCCATAAAGTGATAAcc                                                                                                                                                                                                                      <  1:276734/301‑1 (MQ=255)`  
                                                                                     `cGGTTGACCGTGAGGTTTCCCAGAGGGGTGGGGATTACCCTTGGGTTTGTATTTGGGTGTTACCTTACGTGTTTCTCTGTGT‑AAC‑ATCTGATACAGTCGCCTAAAGCAGCCTCCTCGAGGCACCTCCTAACGTCTATGCTAAAGCCAACACCTACAGCCTAGACATAAAGACCCACCAGGGCAACGCCTATCTTAAAGTTTAAACATAAAGACCAGACCTAAAGACCAGGCCGAAAGACACTAAATAAAGACCAGACCTAAAGACGCCTTGTTGTTAGCCATAAAGTGATAACCTTTAATc                                                                                                                                                                                                               <  1:201426/301‑1 (MQ=255)`  
                                                                                      `tgTTGACCGTGAGGGTTCCCTAAGGGTTGGGGATGACCCTTGGGTTTGTCTTTGGGTGTTACCTTGAGTGTCTCTCTGTGT‑CACTATCTTTAACAGTCTCCTAAAGTATCCTCCTAAAGTCACCTCCTAACGTCCATCCTAAAGACAACACCTAAAGCCTACACCTAAAGACCCATCAAGTCAACGCCTATCTTAAAGTTTAAACATAAAGACCAGACCTAAAGACCAGACCTAAAGACACTACATAAAGACCAGACCTAAAGACGCCTTGTTGTTAGCCATAAAGTGATAACCTTTAATc                                                                                                                                                                                                               <  1:100116/300‑1 (MQ=255)`  
                                                                                       `cggagatgtgtataagagacaggggTTGGGTAAGACCCTTGGGTTTGTCTTTGGGTGTTACCTTGAGTGTCTCTCTGTGTCCCCTATCTGTTACAGTCTCCTAAAGTATCCTCCTAAAGTCACCTCCTAACGTCCATCCTAAAGCCAACACCTAAAGCCTACACCTAAAGACCCATCAAGTCAACGCCTATCTTAAAGTTTAAACATAAAGACCAGACCTAAAGACCAGACCTAAAGACACTACATAAAGACCAGACCTAAAGACGCCTTGTTGTTAGCCATAAAGTGATAACCTTTAATc                                                                                                                                                                                                               <  1:198743/279‑1 (MQ=255)`  
                                                                                        `ttGACCGTGAAGGTTCCCAAAGGGTTGGGGATGACCCTTGGGTTTGTCATTTGGTGGTACCTTGAGTGTCTCTCTGTGTACCCTATCTGTGACAGTCTCCTAAAGTATCCTCCTAAAGACACCGCCTAACGTCCATCCTAAAGCCAACACCTAAAGCCTACACCGAAAGACCCATCAAGTCAACGCCTATCTTAAAGTTTAAACATAAAGACCAGAACTAAAGACCAGACCTAAAGACACTACATAAAGACCAGAACTAAAGACGCCGTGTTGTTAGCCATAAAGTGATAACCTTTAATCa                                                                                                                                                                                                              <  1:189548/301‑1 (MQ=255)`  
                                                                                            `ccTTGAGTGTTCCCTAAGGGTTGGGGATGACCCTTGGGTTTGTCTTTGGGTGTTACCTTGAGTGTCTCTCTGTGTCCCCTATCTGTTACAGTCTCCTAAAGTATCCTCCTAAAGTCACCTCCTAACGTCCATCCTAAAGCCAACACCTAAAGCCTACACCTAAAGACCCATCAAGTCAACGCCTATCTTAAAGTTTAAACATAAAGACCAGACCTAAAGACCAGAACTAAAGACACTACATAAAGACCAAACCTAAAGACGCCTTGTTCTTAGCCATAAAGTACTACCCttcttgcttttt                                                                                                                                                                                                          >  1:310996/1‑291 (MQ=39)`  
                                                                                                     `ttCCCTAAGGGTTGGGGATGACCCTTGGGTTTGTCTTTGGGTGTTACCTTGAGTGTCTCTCTGTGTCCCCTATCTGTTACAGTCTCCTAAAGTATCCTCCTAAAGTCACCTCCTAACGTCCATCCTAAAGCCAACACCTAAAGCCTACACCTAAAGACCCATCAAGTCAACGCCTATCTTAAAGTTTAAACATAAAGACCAGACCTAAAGACCAGACCTAAAGACACTACATAAAGACCAGACCTAAAGACGCCTTGTTGTTAGCCATAAAgctgtctcttatacacatctccgagcccac                                                                                                                                                                                                 >  1:288279/1‑272 (MQ=39)`  
                                                                                                       `cccTAAGGGTTTGGGATGACCCTTGGGTTTGTCTTTGGGTGTTACCTTGAGTGTCTCTCTGTGT‑CCCTATCTGTAACAGTCTCCTAAAGTATCCTCCTAAAGTCATCTCCTAAAGTCCATCCTAAAGCCAACACCTAAAGCCTACACCTAAAGACCCATCAAGTCAACGCCTATCTTAAAGTTTAAACATAAAGACCAGACCTAAAGACCAGACCTAAAGACACTACATAAAGACCAGACCTAAAGACGCCTTGTTGTTAGCCATAAAGTAATAACCTTTAATCATTGTCTTTATTAAtta                                                                                                                                                                                              >  1:207017/1‑299 (MQ=255)`  
                                                                                                       `cccTAAGGGTTGGGGATGACCCTTGGGTTTGTCTTTGGGTGTTACCTTGAGTGTCTCTCTGTGT‑CCCTATCTGTAACAGTCTCCTAAAGTATCCTCCTAAAGTCACCTCCTAACGTCCATCCTAAAGCCAACACCTAAAGCCTACACCTAAAGACCCATCAAGTCAACGCCTATCTTAAAGTTTAAACATAAAGACCAGACCTAAAGACCAGACCTAAAGACACTACATAAATACCAGACCTAAAGACGCCTTGTTGTTAGCCATAAAGTGATAACCTTTAATCATTGTCTTTATTAATAc                                                                                                                                                                                              >  1:232156/1‑301 (MQ=255)`  
                                                                                                       `agacatgggTTGGGGATGACCCTTTGGTTTGTCTTTGTGTGTTACCTTGAGTGTCTCTCTGTGT‑CCCTATCTGTAACAGTCTCCTAAAGTATCCTCCTAAAGTCACCTCCTAACGTCCATCCTAAAGCCAACACCTAAAGCCTACACCTAAAGACCCATCAAGTCAACGCCTATCTTAAAGTTTAAACATAAAGACCAGACCTAAAGACCAGACCTAAAGACACTACATAAAGACCAGACCTAAAGACGCCTTGTTGTTAGCCATAAAGTGATAACCTTTAATCATTGTCTTTATTAATAc                                                                                                                                                                                              <  1:194972/295‑1 (MQ=255)`  
                                                                                                                 `tGGGGTTTACCCTTGGGTTTGTCTTTGGGTGTTACCTTGAGTTTCTCTCTGTTT‑CCCTATCTGTAACAGTCTCCTAAAGTATCCTCCTAAAGTCACCTCCTAACGTCCATCCTAAAGCCAACACCTAAAGCCTACACCTAAAGACCCATCAAGTCAACGCCTACCTTAAAGTTTAAACATAAAGACCAGACCTAAAGACCAGACCTAAAGACACTACATAAAGACCAGACCTAAAGACGCCTTGTTGTTAGCCATAAAGTGATAACCTTTAATCATTGTCTTTATTAATACAACTCACtat                                                                                                                                                                                    <  1:136660/301‑1 (MQ=255)`  
                                                                                                                   `gggATGACCCTTGGGTTTGTCTTTGGTTGTTACCTTGAGTGTCTCTCTGTGT‑CCCTATCTG‑TACAGTCTCCTAAAGTATCCTCCTAAAGTCACCTCCTAACGTCCATCCTAAAGCCAACACCTAAAGCCTACACCTAAAGACCCATCAAGTCAACGCCTATCTTAAAGTTTAAACATAAAGACCAGACCTAAAGACCAGACCTCAAGACACTACATAAAGACCAGACCTAAAGACGCCTTGTTGTTAGCCATAAAGTGATAACCTTTAATCATTGTCTTTATTACTACAACTCACTATAAg                                                                                                                                                                                 >  1:129752/1‑301 (MQ=255)`  
                                                                                                                    `ggATGACCCTTGGGTTTGTCTTTGGGTGTTACCTTGAGTGTCTCTCTGTGT‑CCCTATCTGTAACAGTCTCCTAAAGTATCCTCCTAAAGTCACCTCCTAACGTCCATCCTAAAGCCAACACCTAAAGCCTACACCTAAAGACCCATCAAGTCAACGCCTATCTTAAAGTTTAAACATAAAGACCAGACCTAAAGACCAGACCTAAAGACACTACATAAAGACCAGACCTAAAGACGCCTTGTTGTTAGCCATAAAGTGATAACCTTTAATCATTGTCTTTATTCATAcctgtctcttatac                                                                                                                                                                                 >  1:273132/1‑288 (MQ=255)`  
                                                                                                                    `agacagcccTTGGGTTTGTCTTTGGGTGTTACCTTGAGTGTCTCTCTGTGT‑CCCTATCTGTAACAGTCTCCTAAAGTATCCTCCTAAAGTCACCTCCTAACGTCCATCCTAAAGCCAACACCTAAAGCCTACACCTAAAGACCCATCAAGTCAACGCCTATCTTAAAGTTTAAACATAAAGACCAGACCTAAAGACCAGACCTAAAGACACTACATAAAGACCAGACCTAAAGACGCCTTGTTGTTAGCCATAAAGTGATAACCTTTAATCATTGTCTTTATTAATACAACTCACTATAAg                                                                                                                                                                                 <  1:69240/295‑1 (MQ=255)`  
                                                                                                                     `tgtgtataagagacaggTCTTTTGGTGTTACCTTGAGTGTCTCTCTGTGT‑‑CCTATCTGTTACAGTCTCCTAAAGTATCCTCCTAAAGTCACCTCCTAACGTCCATCCTAAAGCCAACACCTAAAGCCTACACCTAAAGACCCATCAAGTCAACGCCTATCTTAAAGTTTAAACATAAAGACCAGACCTAAAGACCAGACCTAAAGACACTACATAAAGACCAGACCTAAAGACGCCTTGTTGTTAGCCATAAAGTGATAACCTTTAATCATTGTCTTTATTAATACAACTCACTATAAGga                                                                                                                                                                               <  1:75570/285‑1 (MQ=255)`  
                                                                                                                      `aTGACCCTTGGGTTTGTCTTTGGGTGTTACCTTGAGTGTCTCTCTGTGT‑CCCTATCTGTAACAGTCTCCTAAAGTATCCTCCTAAAGTCACCTTCTAACGTCCATCCTAAAGCCAACACCTAAAGCCTACACCTAAAGACCCATCAAGTCAACGCCTATCTTAAAGTTTAAACATAAAGACCAGACCTAAAGACCAGACCTAAAGACACTACATAAAGACCAGACCTAAAGACGCCTTGTTGTTAGCCATAAAGTGATAACCTTTAATCATTGTCTTTATTAATACAACTCACTATAAGga                                                                                                                                                                               <  1:294614/301‑1 (MQ=255)`  
                                                                                                                       `agACGCTGGGGCTCGTCTTTGGGCGTTACCTTTAGTGTCTCTCTGTGTCCCCTATCTGTGACAGTCTCCTAAAGTATCCTTCTAAAGTCACCTCCTAACGTCCATCCTAAAGCCAACACCTAAAGCCTACACCCAAAGACCCATCAAGACAACGCCTATCTTAAAGTTTAAACATAAAGACCAGACCTAAAGACCAGACCTAAAGACACTACATAAAGACCAGACCTAAAGACGCCTTGTTGTTAGCCATAAAGTGATAACCTTTAATCATTGTCTTTATTAATACAACTCACTATAAGga                                                                                                                                                                               <  1:303800/300‑1 (MQ=255)`  
                                                                                                                        `gtataagagacaggtcatggggTGTTACCTTGAGTGTCTCTCGACGT‑CCC‑AACTGTTACAGTCTCCTAAAGTATCCTCCTAAAGTCACCTCCTAACGTCCATCCTAAAGCCAACACCTAAAGCCTACACCGAAAGACCCATCAAGTCAACGCCGATCTTAAAGTTTAAACATAAAGACCAGACCTAAAGACCAGACCTAAAAACACTACATAAAGACCAGACCTAAAGACGACTTGTTGTTAGCCATAAAGTGATAACCTTTAATCATTGTCTTTATTAATACAACTCACTATAAGgagag                                                                                                                                                                            <  1:285939/282‑1 (MQ=255)`  
                                                                                                                          `cccTTTGGTTTGTCTTTGGGTGTTACCTTGAGTGTCTCTCTGTGT‑CCCTATCTG‑TACAGTCTCCTAAAGTATCCTCCTAAAGTCACCTCCTAACGTCCATCCTAAAGCCAACACCTAAAGCCTACACCTAAAGACCCATCAAGTCAACGCCTATCTTAAAGTTTAAACATAAAGACCAGACCTAAAGACCAGACCTAAAGACACTACATAAAGACAAGACCTAAAGACGCCTTGTTGTTAGCCATAAAGTGATAACCTTTAATCATTGTCTTTATTAATACAACTCACTATACGGAGAGAc                                                                                                                                                                          >  1:45939/1‑301 (MQ=255)`  
                                                                                                                          `cccTTGGGTTTGTCTTTGGGTGTTACCTTGAGTGTCTCTCTGTGT‑CCCTATCTGTAACAGTCTCCTAAAGTATCCTCCTAAAGTCACCTCCTAACGTCCATCCTAAAGCCAACACCTAAAGCCTACACCTAAAGACCCATCAAGTCAACGCCTATCTTAAAGTTTAAACATAAAGACCAGACCTAAAGACCAGACCTAAAGACAATACATAAAGACCAGACCTAAAGACGCCTTGTTGTTAGCCATAAAGTGATAACCTTTAATCATTGTCTTTATTAATACAACTCACTATAAGgactgt                                                                                                                                                                           >  1:12366/1‑297 (MQ=255)`  
                                                                                                                                     `gTCTTTGGGTGTTACCTTGAGTGTCTCTCTGTGT‑CCCTATCTGTAACAGTCTCCTAAAGTATCCTCCTAAAGTCACCTCCTAACGTCCATCCTAAAGCCAACACCTAAAGCCTACACCTAAAGACCCATCAAGTCAACGCCTATCTTAAAGTTTAAACATAAAGACCAGACCTAAAGACCAGACCTAAAGACACTACATAAAGACCAGACCTAAAGACGCCTTGTTGTTAGCCATAAAGTGATAACCTTTAATCATTGTCTTTATTAATACAACTCACTATAAGGAGAGACAACTTAAaga                                                                                                                                                                >  1:115123/1‑301 (MQ=255)`  
                                                                                                                                       `cTTTGGGTGTTACCTTGAGTGTCTCTCTGTGT‑CCCTATCTGTAACAGTCTCCTAAAGTATCCTCCTAAAGTCACCTCCTAACGTCCATCCTAAAGCCAACACCTAAAGCCTACACCTAAAGACCCATCAAGTCAACGCCTATCTTAAAGTTTAAACATAAAGACCAGACCTAAAGACCAGACCTAAAGACACTACATAAAGACCAGACCTAAAGACGCCTTGTTGTTAGCCATAAAGTGATAACCTTTAATCATTGTCTTTATTAATACAACTCACTATAAGGAGAGAcctgtctcttata                                                                                                                                                              >  1:329342/1‑289 (MQ=255)`  
                                                                                                                                             `gtgtTACCTTGAGTGTCTCTCTGTGT‑CCCTATCTGTAACAGTCTCCTAAAGTATCCTCCTAAAGTCACCTCCTAACGTCCATCCTAAAGCCAACACCTAAAGCCTACACCTAAAGACCCATCAAGTCAACGCCTATCTTAAAGTTTAAACATAAAGACCAGACCTAAAGACCAGACCTAAAGAAACTACATAAAGACCAGACCTAAAGACGCCTTGTTGTTAGCCATAAAGTGATAACCTTTAATCATTGTCTTTATTAATACAACTCACTATAAGGAGAGACAACTTAAAGAGACTTaaa                                                                                                                                                        >  1:37845/1‑301 (MQ=255)`  
                                                                                                                                                `ttACCTTGAGTGTCTCTCTTAGTCCCCTATCTGTTATAGTCTCCTAAAGTATCCGCCTAAAGTCACCTCCTAACGTCCATCCTAAAGCCAACACCTAAAGCCTACACCGAAAGACCCATCAAGTCAACGCCGATCTTAAAGTTTAAACATAAAGACCATACCTAAAGACTAGACCTAAAGACACTACATAAAGACCAGACCTAAAGACGCCTTGTTGTTGGCCATAAAGTGAGAACCTTTAATCATTGTCTTTATTAATACAACTCACTATAAGGAGAGACAACTTAAAGAGACTTAAAAg                                                                                                                                                      <  1:271274/301‑1 (MQ=255)`  
                                                                                                                                                  `aCCTTTAGTGTCTCTCTGTGT‑CCC‑ATCTGTTACAGTCTCCTAAAGTATCCTCCTAAAGTCACCTCCTAACGTCCATCCTAAAGCCAACACCTAAATCCTACACCTAAAGACCCATCAAGTCAACGCCTATCTTAAAGTTTAAACATAAAGACCAGACCTAAAGACCATACCTAAAGACACTACATAAAGACCAGACCTAAAGACGCCTTGTTGTTAGCCATAAAGTGATAACCTTTAATCATTGTCTTTATTAATACAACTCACTATAAGGAGAGACAACTTAAagctgtctcttatacac                                                                                                                                                  >  1:224584/1‑286 (MQ=255)`  
                                                                                                                                                           `gtCTCTCTGTGT‑CCCTATCTG‑TACAGTCTCCTAAAGTATCCTCCTAAAGTCACCTCCTAACGTCCATCCTAAAGCCAACACCTAAAGCCTACACCTAAAGACCCATCAAGTCAACGCCTATCTTAAAGTTTAAACATAACGACCAGACCTAAAGACCAGACCTAAAGACACTACATAAAGACCAGACCTTAAGACGCCTTGTTGTTAGCCATAAAGTGATAACCTTTAATCATTGTCTTTATTAATACAACTCACTATAAGGAGAGACAACTTAAAGAGACTTAAAAGATTAATTTAAAAt                                                                                                                                         >  1:252428/1‑301 (MQ=255)`  
                                                                                                                                                                            `aTCTGTTACAGTCTCCTAAAGTATCCTCCTAAAGTCACCTCCTAACGTCCATCCTAAAGCCAACACCTAAAGCCTACACCTAAAGACCCATCAAGTCAACGCCTATCTTAAAGTTTAAACATAAAGACCAGACCTAAAGACCAGACCTAAAGACACTACATAAAGACCAGACCTAAAGACGCCTTGTTGTTAGCCATAAAGTGATAACCTTTAATCATTGTCTTTATTAATACAACTCACTATAAGGAGAGACAACTTAAAGAGACTTAAAAGATTAATTTAAAATTTATCAAAAAGAGTa                                                                                                                          >  1:17457/1‑301 (MQ=255)`  
                                                                                                                                                                            `aTCTGTAACAGTCTCCTAAAGTATCCTCCTAAAGTCACCTCCTAACGTCCATCCTAAAGCCAACACCTAAAGCCTACACCTAAAGACCCATCAAGTCAACGCCTATCTTAAAGTTTAAACATAAAGACCAGACCTAAGGACCAGACCTAAAGACACTACATAAAGACCAGACCTAAAGACGCCTTGTTGTTAGCCATAAAGTGATAACCTTTAATCATTGTCTTTATTAATACAACTCACTATAAGGAGAGACAACTTAAAGAGACTTAAAAGATTAATTTAAAATTTATCAAAAAGAGTa                                                                                                                          >  1:140578/1‑301 (MQ=255)`  
                                                                                                                                                                            `aTCTGTAACAGTCTCCTAAAGTATCCTCCTAAAGTCACCTCCTAACGTCCATCCTAAAGCCAACACCTAAAGCCTACACCTAAAGACCCATCAAGTCAACGCCTATCTTAAAGTTTAAACATAAAGACCAGACCTAAAGACCAGACCTAAAGACACTACATAAAGACCAGACCTAAAGACGCCTTGTTGTTAGCCATAAAGTGATAACCTTTAATCATTGTCTTTATTAATACAACTCACTATAAGGAGAGACAACTTAAAGAGACTTAAAAGATTAATTTAAAATTTATCAAAAAGAGTa                                                                                                                          >  1:222158/1‑301 (MQ=255)`  
                                                                                                                                                                            `aTCTGTAACAGTCTCCTAAAGTATCCTCCTAAAGTCACCTCCTAACGTCCATCCTAAAGCCAACACCTAAAGCCTACACCTAAAGACCCATCAAGTCAACGCCTATCTTAAAGTTTAAACATAAAGACCAGACCTAAAGACCAGACCTAAAGACACTACATAAAGACCAGACCTAAAGACGCCTTGTTGTTAGCCATAAAGTGATAACCTTTAATCATTGTCTTTATTAATACAACTCACTATAAGGAGAGACAACTTAAAGAGACTTAAAAGATTAATTTAAAATTTATCAAAAAGAGTa                                                                                                                          >  1:204106/1‑301 (MQ=255)`  
                                                                                                                                                                             `tCTGTTACAGTCTCCTAAAGTATCCTCCTAAAGTCACCTCCTAACGTCCATCCTAAAGCCAACACCTAAAGCCTACACCTAAAGACCCATCAAGTCAACGCCTATCTTAAAGTTTAAACATAAAGACCAGACCTAAAGACCAGACCTAAAGACACTACATAAAGACCAGACCTAAAGACGCCTTGTTGTTAGCCATAAAGTGATAACCTTTAATCATTGTCTTTATTAATACAACTCACTATAAGGAGAGACAACTTAAAGAGACTTAAAAGATTAATTTAAAATTTATCAAAAAGAGTAt                                                                                                                         <  1:43059/301‑1 (MQ=255)`  
                                                                                                                                                                              `cTGTAACAGTCTCCTAAAGTATCCTCCTAAAGTCACCTCCTAACGTCCATCCTAAAGCCAACACCTAAAGCCTACACCTAAAGACCCATCAAGTCAACGCCTATCTTAAAGTTTAAACATAAAGACCAGACCTAAAGACCAGACCTAAAGACACTACATAAAGACCAGACCTAAAGACGCCTTGTTGTTAGCCATAAAGTGATAACCTTTAATCATTGTCTTTATTAATACAACTCACTATAAGGAGAGACAACTTAAAGAGACTTAAAAGATTAATTTAAAATTTATCAAAAAGAGTAtt                                                                                                                        >  1:8352/1‑301 (MQ=255)`  
                                                                                                                                                                                 `gagatgtggataagagacagctcctAAAGTCACCTCCTAACGTCCATCCTAAAGCCAACACCTAAAGCCTACACCTAAAGACCCATCAAGTCAACGCCTATCTTAAAGATTAAACATAAAGACCAGACCTAAAGACCAGACCTAAAGACACTACATAAAGACCAGACCTAAAGACGCCTTGTTGTTAGCCATAAAGTGATAACCTTTAATAATTGTCTTTATTAATACAACACACTATAAGGAGAGACAACTTAAAGAGACTTAAAAGATTAATTTAAAATTTCTCAAAAAGAGTATTGAc                                                                                                                     <  1:170804/281‑1 (MQ=255)`  
                                                                                                                                                                                       `gtgtataaaagacagtcctAGAGAAACCTCCTAACGTCCAACCTAAAGCCAACACCTAAAGCCTACACCTAAAGACCCATCAAGTCAACGCCTATCTTAAAGATTAAACATAAAGACCAGACCTAAAGACGAGACCTAAAGACACTACATAAAGACCAGACCTAAAGACGCCTTGTTGTTAGCAATAAAGTGATAACCTTTAATCATTGTCTTTATTAATACAACTCAATATAAGGAGAGACAACTTAAAGAGACTTAAAAGATTAATTTAAAATTTATCAAAAAGAGTATTGACTTAAAg                                                                                                               <  1:335562/286‑1 (MQ=255)`  
                                                                                                                                                                                        `ctcCTAAAGTATCCTCCTAAAGTCACCTCCTAACGTCCATCCTAAAGCCAACACCTAAAGCCTACACCTAAAGACCCATCAAGTCAACGCCTATCTTAAAGTTTAAACATAAAGACCAGACCTAAAGACCAGACCTAAAGACACTACATAAAGACCAGACCTAAAGACGCCTTGTTGTTAGCCATAAAGTGATAACCTTTAATCATTGTCTTTATTAATACAACTCACTATAAGGAGAGACAACTTAAAGAGACTTAAAAGATTAATTTAAAATTTATCAAAAAGAGTATTGACTTAAAGt                                                                                                              >  1:268204/1‑301 (MQ=255)`  
                                                                                                                                                                                        `ctcCTAAAGTATCATCCTAAAGTCACCTCCTAACGTCCATCCTAAAGCCAACACCTAAAGCCTACACCTAAAGACCCATCAAGTCAACGACTATCTTAAAGTTTAAACATAAATACCAGACCTAAAGACCAGACCTAAAGACACTACATAAAGACCAGACCTAAAGACGCCTTGTTGTTAGCCATAAATTGTTAACCTTTAATCATTGTCTTTATTAATACAACTAACTATAAGGAGCGACAACTTAAATAGACTTAACAGATTAATTTAAAATTTATCAAAAAGAGTATTGACTTAAAGt                                                                                                              >  1:228466/1‑301 (MQ=255)`  
                                                                                                                                                                                         `tcCTAAAGTATCCTCCTAAAGTCACCTCCTAACTTCCAACCTAAAGCCAACACCTAAAGCCTACACCTAAAGACCCATCAAGTCAACGCCTATCTTAAAGTTTAAACATAAAGACCAGACCTAAATACCAGACCTAAAGACACTACATAAAGACCAGACCTAAAGACGCCTTGTTGTTAGCCATAAAGTGATAACCTTTAATCATTGTCTTTATTAATACAACTCACTATAAGGAGAGACAACTTAAAGAGACTTAAAAGATTAATTTAAAATTTATCAAAAAGAGTATTGACTTAAAGTc                                                                                                             <  1:152865/301‑1 (MQ=255)`  
                                                                                                                                                                                          `ccTAAAGTATCCTCCTAAAGTCACCTCCTAACGTCCATCCTAAAGCCAACACCTAAAGCCTACACCTAAAGACCCATCAAGTCAACGCCTATCTTAAAGTTTAAACATAAAGACCAGACCTAAAGACCAGACCTAAAGACACTACATAAAGACCAGACCTAAAGACGCCTTGTTGTTAGCAATAAAGTGATAACCTTTAATCATTGTCTTTATTAATACAACTCACTATAAGGAGAGACAACTTAAAGAGACTTAAAAGATTAATTTAAAATTTATCAAAAAGAGTATTGACTTAAAGTCt                                                                                                            >  1:163077/1‑301 (MQ=255)`  
                                                                                                                                                                                             `aaaGTATCCTCCTAAAGTCACCTCCTAACGTCCATCCTAAAGCCAACACCTAAAGCCTACACCTAAAGACCCATCAAGTCAACGCCTATCTTAAAGTTTAAACATAAAGACCAGACCTAAAGACCAGACCTAAAGACACTACAAAAAGACCAGACCTAAAGACGCCTTGTTGTTAGCCATAAAGTGATAACCTTTAATCATTGTCTTTATTAATACAACTCACTATAAGGAGAGACAACTTAAAGAGACTTAAAAGATTAATTTAAAATTTATCAAAAAGAGTATTGACTTAAAGTCTAAc                                                                                                         <  1:184255/301‑1 (MQ=255)`  
                                                                                                                                                                                              `aaGTATCCTCCTAAAGTCACCTCCTAACGTCCATCCTAAAGCCAACACCTAAAGCCTACACCTAAAGACCCATCAAGTCAACGCCTATCTTAAAGTTTAAACATAAAGACCAGACCTAAAGACCAGACCTAAAGACACTACATAAAGACCAGACCTAAAGACGCCTTGTTGTTAGCCATAAAGTGATAACCTTTAATCATTGTCTTTATTAATACAACTCACTATAAGGAGAGACAACTTAAAGAGACTTAAAAGATTAATTTAAAATTTATCAAAAAGAGTATTGACTTAAAGTCTAAcc                                                                                                        <  1:212705/301‑1 (MQ=255)`  
                                                                                                                                                                                              `aaGTATCCTCCTAAAGTCACCTCCTAACGTCCATCCTAAAGCCAACACCTAAAGCCTACACCTAAAGACCCATCAAGTCAACGCCTATCTTAAAGTTTAAACATAAAGACCAGACCTAAAGACCAGACCTAAAGACACTACATAAAGACCAGACCTAAAGACGCCTTGTTGTTAGCCATAAAGTGATAACCTTTAATCATTGTCTTTATTAATACAACTCACTATAAGGAGAGACAACTTAAAGAGACTTAAAAGATTAATTTAAAATTTATCAAAAAGAGTATTGACTTAAAGTCTAAcc                                                                                                        <  1:242823/301‑1 (MQ=255)`  
                                                                                                                                                                                               `agacagctcctAAAGTCACCTCCTAACGTCCATCCTAACGCCAACACCTAAAGCCTACACCTAAAGACCCATCAAGTCAACGCCTATCTTAAAGTTTAAACATAAAGACCAGACCTAAAGACCAGACCTAAAGACACTACATAAAGACCAGACCTAAAGACGCCTTGTTGTTAGCCATAAAGTGATAACCTTTAATCATTGTCTTTATTAATACAACTCACTATAAGGAGAGACAACTTAAAGAGACTTAAAAGATTAATTTAAAATTTATCAAAAAGAGTATTGACTTAAAGTCTAACCt                                                                                                       <  1:120978/295‑1 (MQ=255)`  
                                                                                                                                                                                                     `ctcctAAAGTCACCTCCTAACGTCCATCCTAAAGCCAACACCTAAAGCCTACACCTAAAGACCCATCAAGTCAACGCCTATCTTAAAGTTTAAACATAAAGACCAGACCTAAAGACCAGACCTAAAGACACTACATAAAGACCAGACCTAAAGACGCCTTGTTGTTAGCCATAAAGTGATAACCTTTAATCATTGTCTTTATTAATACAACTCACTATAAGGAGAGACAACTTAAAGAGACTTAAAAGATTAATTTAAAATTTATCAAAAAGAGTATTGACTTAAAGTCTAACCTATAGGa                                                                                                 >  1:249815/1‑301 (MQ=255)`  
                                                                                                                                                                                                       `cctAAAGTCACCTCCTAACGTCCATCCTAAAGCCAACACCTAAAGCCTACACCTAAAGACCCATCAAGTCAACGCCTATCTTAAAGTTTAAACATAAAGACCAGACCTAAAGACCAGACCTAAAGACACTACATAAAGACCAGACCTAAAGACGCCTTGTTGTTAGCCATAAAGTGATAACCTTTAATCATTGTCTTTATTAATACAACTCACTATAAGGAGAGACAACTTAAAGAGACTTAAAAGATTAATTTAAAATTTATCAAAAAGAGTATTAACTTAAAGTCTAACCTATAGGaat                                                                                               >  1:49213/1‑299 (MQ=255)`  
                                                                                                                                                                                                               `cACCTCCTAACGTCCATCCTAAAGCCAACACCTAAAGCCTACACCTAAAGACCCATCAAGTCAACGCCTATCTTAAAGTTTAAACATAAAGACCAGACCTAAAGACCAGACCTAAAGACACTACATAAAGACCAGACCTAAAGACGCCTTGTTGTTAGCCATAAAGTGATAACCTTTAATCATTGTCTTTATTAATACAACTCACTATAAGGAGAGACAACTTAAAGAGACTTAAAAGATTAATTTAAAATTTATCAAAAAGAGTATTGACTTAAAGTCTAACCTATAGGATACTTACAGc                                                                                       >  1:297071/1‑301 (MQ=255)`  
                                                                                                                                                                                                                `ccagcctAACGTCCATCCTAAAGCCAACACCTAAAGCCTACACCTAAAGACCCATCAAGTCAACGCCTATCTTAAAGTTTGAACATAAAGACCAGACCTAAAGACCAGACCTAAAGACACTACATAAAGACCAGACCTAAAGACGCCTTGTTGTTAGCCATAAAGTGATAACCTTTAATCATTGTCTTTATTAATACAACTCACTATAAGGAGAGACAACTTAAAGAGACTTAAAAGATTAATTTAAAATTTATCAAAAAGAGTATTGACTTAAAGTATAACCTATAGGATACTTACAGcc                                                                                      <  1:235744/297‑1 (MQ=255)`  
                                                                                                                                                                                                                `accaccTAACGTCCAGCCTAAAGCCAACACCTAAAGCCTACACCTACAGACCCATCAAGTCAACGCCTATCTTAAAGTTTAAACATAAAGACCAGACCTAAAGACCAGACCTAAAGACACTACATAAAGACCATACCTAAAGACGCCTTGTTGTTAGCCATAAAGTGATAACCTTTAATCATTGTCTTTATTAATACAACTCACTATAAGGAGAGACAACTTAAAGAGACTTAAAAGATTAATATAAAATTTATCAAAAATAGTATTGACTTAAAGTCTAACCTATAGGATACTTACAGcc                                                                                      <  1:160/301‑1 (MQ=255)`  
                                                                                                                                                                                                                 `cctcctAACGTCCATCCTAAAGCCAACACCTAAAGCCTACACCTAAAGACCCATCAAGTCAACGCCTATCTTAAAGTTTAAACATAAAGACCAGACCTAAAGACCAGACCTAAAGACACTACATAAAGACCAGACCTAAAGACGCCTTGTTGTTAGCCATAAAGTGATAACCTTTAATCATTGTCTTTATTAATACAACTCACTATAAGGAGAGACAACTTAAAGAGACTTAAAAGATTAATTTAAAATTTATCAAAAAGAGTATTGACTTAAAGTCTAACCTATAGGATACTTACAGCCa                                                                                     >  1:325858/1‑301 (MQ=255)`  
                                                                                                                                                                                                                 `cctcctAACGTCCATCCTAAAGCCAACACCTAAAGCCTACACCTAAAGACCCATCAAGTCAACGCCTATCTTAAAGTTTAAACATAAAGACAAGACCTAAAGACCAGACCTAAAGACACTACATAAAGACCAGACCTAAAGACGCCTTGTTGTTAGCCATAAAGTGATAACCTTTAATCATTGTCTTTATTAATACAACTCACTATAAGGAGAGACAACTTAAAGATACGTAAAAGCTTAATTTAAAATTTATCAAAAAGAGTATTAACTTAACGTCTAACCTATAGCAAACTTACAGcaa                                                                                     >  1:96861/1‑299 (MQ=255)`  
                                                                                                                                                                                                                    `cctAACGTCCATCCTAAAGCCAACACCTAAAGCCTACACCTAAAGACCCATCAAGTCAACGCCTATCTTAAAGTTTAAACATAAAGACCAGACCTAAAGACCAGACCTAAAGACACTACATAAAGACCAGACCTAAAGACGCCTTGTTGTTAGCCATAAAGTGATAACCTTTAATCATTGTCTTTATTAATACAACTCACTATAAGGAGAGACAACTTAAAGAGACTTAAAAGATTCATTTAAAATTTATCAAAAAGAGTATTGACTTAAAGTCTAACCTATAGGATACTTACAGCCATCg                                                                                  >  1:305639/1‑301 (MQ=255)`  
                                                                                                                                                                                                                         `cGTCCATCCTAAAGCCAACACCTAAAGCCTACACCTAAAGACCCATCAAGTCAACGCCTATCTTAAAGTTTAAACATAAAGACCAGACCTAAAGACCAGACCTAAAGACACTACATAAAGACCAGACCTAAAGACGCCTTGTTGTTAGCCATAAAGTGATAACCTTTAATCATTGTCTTTATTAATACAACTCACTATAAGGAGAGACAACTTAAAGAGACTTAAAAGATTAATTTAAAATTTATCAAAAAGAGTATTGACTTAAAGTCTAACCTATAGGATACTTAcctgtctcttataa                                                                             >  1:92275/1‑288 (MQ=255)`  
                                                                                                                                                                                                                         `cGTACATCCTAAAGCCAACACCTAAAGCCTACACCTAAAGACCCATCAAGTCAACGCCTATCTTAAAGTTTAAACATAAAGACCAGACCTAAAGACCAGACCTAAAGACACTACATAAAGACCAGACCTAAAGACGCCTTGTTGTTAGCCATAAAGTGATAACCTTTAATCATTGTCTTTATTAATACAACTCACTATAAGGAGAGACAACTTAAAGAGACTTAAAAGATTAATTTAAAATTTATCAAAAAGAGTATTGACTTAAAGTCTAACCTATAGGATACTTACAGCCATCGAGAgg                                                                             <  1:90019/301‑1 (MQ=255)`  
                                                                                                                                                                                                                          `gTCCATCCTAAAGCCAACACCTAAAGCCTACACCTAAAGACCCATCAAGTCAACGCCTATCTTAAAGTTTAAACATAAAGACCAGACCTAAAGACCAGACCTAAAGACACTACATAAAGACCAGACCTAAAGACGCCTTGTTGTTAGCCATAAAGTGATAACCTTTAATCATTGTCTTTATTAATACAACTCACTATAAGGAGAGACAACTTAAAGAGACTTAAAAGATTAATTTAAAATTTATCAAAAAGAGTATTGACTTAAAGTCTAACCTATAGGATACTTACAGCCATCGAGAggg                                                                            >  1:280621/1‑301 (MQ=255)`  
                                                                                                                                                                                                                                `ccTAAATCCAACACCTAAATCCTACACCTAAATACCCATCAAGTCAACGTCTATCTTAAAGTTTAAATATAAAGACCAGACCGAAAGACCAGACCTAAAGACACTACATAAAGACCAGACCTAAAGACGCCTTGTTGTTAGCCATAAAGTGATAACCTTTAATCATTCTCTTTATTAATACAACTCACTATAATGAGAGACAACTTAAAGAGACTTAAAAGATTAATTTAAAATTTATCAAAAAGAGTATTGACTTAAAGTCTAACCTATAGGATACTTACAGCCATCGAGAGGGACACgg                                                                      <  1:180412/301‑1 (MQ=255)`  
                                                                                                                                                                                                                                             `ccTAAAGCCTACACCT‑AAGACCCATCAAGTCAACGCCTATCTTAAAGTTTAAACATAAAGACCAGACCTAAAGACCAGACCTAAAGACACTACATAAAGACCAGACCTAAAGACGCCTTGTTGTTAGCCATAAAGTGATAACCTTTAATCATTGTCTTTATTAATACAACTCACTATAAGGAGAGACAACTTAAAGAGACTTAAAAGATTAATCTAAAATTTATCAAAAAGAGTATTGACTTAAAATCTAACCTATAGTATACTTACAGCCATCGAGAGGGACACGGCTAATAGCCATccc                                                        >  1:168400/1‑301 (MQ=255)`  
                                                                                                                                                                                                                                                   `gCCTACACCTAAAGACCCATCAAGTCAACGCCTATCTTAAAGTTTAAACATAAAGACCAGACCTAAAGACCAGACCTAAATACACTACATAAAGACCAGACCTAAAGACGCCTTGTTGTTAGCCATAAAGTGATAACCTTTAATCATTGTCTTTATTAATACAACTCACTATAAGGAGAGACAACTTAAAGAGACTTAAAAGATTAATTTAAAATTTATCAAAAAGAGTATTGACTTAAAGTCTAACCTATAGGATACTTACAGCCATCGAGAGGGACACGGCGAATAGCCATCCCAATCg                                                   >  1:133144/1‑301 (MQ=255)`  
                                                                                                                                                                                                                                                   `gCCTACACCTAAAGACCCATCAAGTCAACGCCTATCTTAAAGTTTAAACATAAAGACCAGACCTAAAGACCAGACCTAAAGACACTACATAAAGACCAGACCTAAAGACGCCTTGTTGTTAGCCATAAAGTGATAACCTTTAATCATTGTCTTTATTAATACAACTCACTATAAGGAGAGACAACTTAAAGAGACTTAAAAGATTAATTTAAAATTTATCAAAAAGAGTATTGACTTAAAGTCTAACCTATAGGATACTTACAGCCATCGAGAGGGACACGGCGAATAGCCATCCCAATCg                                                   >  1:192795/1‑301 (MQ=255)`  
                                                                                                                                                                                                                                                        `agacagaaGACCCATCAAGTCAACGCCTATCTTAAAGTTTAAACATAAAGACCAGACCTAAAGACCAGACCTAAAGACACTACATAAAGACCAGACCTAAAGACGCCTTGTTGTTAGCCATAAAGTGATAACCTTTAATCATTGTCTTTATTAATACAACTCACTATAAGGAGAGACAACTTAAAGAGACTTAAAAGATTAATTTAAAATTTATCAAAAAGAGTATTGACTTAAAGTCTAACCTATAGGATACTTACAGCCATCGAGAGGGACACGGCGAATAGCCATCCCAATCGACAcc                                              <  1:219016/295‑1 (MQ=255)`  
                                                                                                                                                                                                                                                          `ccTAAAGACCCATCAAGTCAACGCCTATCTTAAAGTTTAAACATAAAGACCAGACCTAAAGACCAGACCTAAAGACACTACATAAAGACCAGACCTAAAGACGCCTTGTTGTTAGCCATAAAGTGATAACCTTTAATCATTGTCTTTATTAATACAACTCACTATAAGGAGAGACAACTTAAAGAGACTTAAAAGATTAATTTAAAATTTATCAAAAAGAGTATTGACTTAAAGTCTAACCTATAGGATACTTACAGCCATCGAGATGGACACGGCGAATAGCCATCCCAATCGACACCgg                                            >  1:86947/1‑301 (MQ=255)`  
                                                                                                                                                                                                                                                          `ccTAAAGACCCATCAAGTCAACGCCTATCTTAAAGTTTAAACATAAAGACCAGACCTAAAGACCAGACCTAAAGACACTACATAAAGACCAGACCTAAAGACGCCTTGTTGTTAGCCATAAAGTGATAACCTTTAATCATTGTCTTTATTAATACAACTCACTATAAGGAGAGACAACTTAAAGAGACTTAAAAGATTAATTTAAAATTTATCAAAAAGAGTATTGACTTAAAGTCTAACCTATAGGATACTTACAGCCATCGAGAGGGACACGGCGAATAGCCATCCCAATCGACACCgg                                            >  1:94356/1‑301 (MQ=255)`  
                                                                                                                                                                                                                                                          `ccTAAAGACCCATCAAGTCAACGCCTATCTTAAAGTTTAAACATAAAGACCAGACCTAAAGACCAGACCTAAAGACACTACATAAAGACCAGACCTAAAGACGCCTTGTTGTTAGCCATAAAGTGATAACCTTTAATCATTGTCTTTATTAACACAACTCACTATAAGGAGAGACAACTTAAAGAGACTTAAAAGATTAATTTAAAATTTATCAAAAAGAGTATTGACTTAAAGTCTAACCTATAGGATACTTACAGCCATCGAGAGGGACACGGAGAATAGCCATCCCAATcctatctct                                            >  1:332743/1‑293 (MQ=255)`  
                                                                                                                                                                                                                                                          `ccTAAAGACCCATCAAGTCAACGCCTATCTTAAAGTTTAAACATAAAGACCAGACCTAAAGACCAGACCTAAAGACACTACATAAAGACCAGACCTAAAGACGCCTTGTTGTTAGCCACAAAGTGATAACCTTTAATCATTGTCTTTATTAATACAACTCACTATAAGGAGAGACAACTTAAAGAGACTTAAAAGATTAATTTAAAATTTATCAAAAAGAGTATTGACTTAAAGTCTAACCTATAGGATACTTACAGCCATCGAGAGGGACACGGCGAATAGCCATCCCAATCGACACCgg                                            >  1:144062/1‑301 (MQ=255)`  
                                                                                                                                                                                                                                                            `aaaaGACCCATCAAGTCAACGCCTATCTTAAAGTTTAAACATAAAGACCAGACCTAAAGACCAGACCTAAAGACACTACATAAAGACCAGACCTAAAGACGCCTTGTTGTTAGCCATAAAGTGATAACCTTTAATCATTGTCTTTATTAATACAACTCACTATAAGGAGAGACAACTTAAAGAGACTTAAAAGATTAATTTAAAATTTATCAAAAAGAGTATTGACTTAAAGTCTAACCTATAGGATACTTACAGCCATCGAGAGGGACACGGCGAATAGCCATCCCAATCGACACCgggg                                          <  1:342516/300‑1 (MQ=255)`  
                                                                                                                                                                                                                                                             `aaaGACCCATCAAGGCAACGCCTATCTTAAAGTTTAAACATAAAGACCAGACCTAAAGACCAGACCTAAAGACACTACATAAAGACCAGACCTAAAGACGCCTTGTTGTTAGCCATAAAGTGATAACCTTTAATCATTGTCTTTATTAATACAACTCACTATAAGGAGAGACAACTTAAAGAGACTTAAAAGATTAATTTAAAATTTATCAAAAAGAGTATTGACTTAAAGTCTAACCTATAGGATACTTACAGCCATCGAGAGGGACACGGCGAATAGCCATCCCAATCGACAccctcac                                         <  1:66310/301‑6 (MQ=255)`  
                                                                                                                                                                                                                                                                  `cccATCAAGTCAACGCCTATCTTAAAGTTTAAACATAAAGACCAGACCTAAAGACCAGACCTAAAGACACTACATAAAGACCAGACCTAAAGACGCCTTGTTGTTAGCCATAAAGTGATAACCTTTAATCATTGTCTTTATTAATACAACTCCCTATAAGGAGAGACAACTTAAAGAGACTTAAAAGATTAATTTAAAATTTATCAAAAAGAGTATTGAATTAAAGTCTAACCTATAGGATACTTACAGCCATCGAGAGGGACACGGCGAATAGCCATCCCAATCGACACCGGGGTCaatc                                    >  1:73057/1‑299 (MQ=255)`  
                                                                                                                                                                                                                                                                       `gacctaaagaccagacctaaagacactaCATAAAGACCAGACCTAAAGACCAGACCTAAAGACACTACATAAAGACCAGACCTAAAGACGCCTTGTTGTTAGCCATAAAGTGATAACCTTTAATCATTGTCTTTATTAATACAACTCACTATAAGGAGAGACAACTTAAAGAGACTTAAAAGATTAATTTAAAATTTATCAAAAAGAGTATTGACTTAAAGTCTAACCTATAGGATACTTACAGCCATCGAGAGGGACACGGCGAATAGCCATCCCAATCGACACCGGGGTCAACCGGATa                               >  1:105949/28‑301 (MQ=255)`  
                                                                                                                                                                                                                                                                              `aCGTCTATCTTAAAGTTTAAACATAAAAACCAGACCTAAAGACCAGACCTAAAGACACTACATAAAGACCAGACCTAAAGACGCCTTGTTGTTAGCCATAAAGTGATAACCTTTAATCATTGTCTTTATTAATACAACTCACTATAAGTAGAGACAACTTAAAGAGACTTAAAAGATTAATTTAGAATTTATCAAAAAGAGTATTGACTTAAAGTCTAACCTATAGGATACTTACAGCCATCGAGAGGGACACGGCGAATAGCCATCCCAATCGACACCGGGGTCAACCGGATAAGTAGAc                        <  1:255070/301‑1 (MQ=255)`  
                                                                                                                                                                                                                                                                              `aCGCCTATCTTAAAGTTTAAACATAAAGACCAGACCTAAAGACCAGACCTAAATACACTACATAAAGACCAGACCTAAAGACGCCTTGTTGTTAGCCATAAAGTGATAACCTTTAATCATTGTCTTTATTAATACAACTCACTATAAGGAGAGACAACTTAAAGAGACTTAAAAGATTAATTTAAAATTTATCAAAAAGAGTATTGACTTAAAGTCTAACCTATAGGATACTTACAGCCATCTAGAGGGACACGGCGAATAGCCATCCCAATCGACACCGGGGTCAACCGGATAAGTAGAc                        <  1:150325/301‑1 (MQ=255)`  
                                                                                                                                                                                                                                                                              `aCGCCTATCTTAAAGTTTAAACATAAAGACCAGACCTAAAGACCAGACCTAAAGACACTACATAAAGACCAGACCTAAAGACGCCTTGTTGTTAGCCATAAAGTGATAACCTTTAATCATTGTCTTTATTAATACAACTCAATATAAGGAGAGACAACTTAAAGAGACTTAAAAGATTAATTTAAAATTTATCAAAAAGAGTATTGACTTAAAGTCTAACCTATAGGATACTTACAGCCATCGAGAGGGACACGGCGAATAGCCATCCCAATCGACACCGGGGTCAACCGGATAAGTAGAc                        <  1:209775/301‑1 (MQ=255)`  
                                                                                                                                                                                                                                                                                  `cTAGCTTAAAGTTTAAACATAAAGACCAGACCTAAAGACCAGACCTAAAGACACGACATAAAGACCAGACCTAAAGACGCCTTGTTGTTAGCCATAAAGTGATAACCTTTAATCATTGTATGTATTAATACAACTCACTATAAGGAGAGACAACTTAAAGAGACTTAAAAGATTAATTTAAAATTTATCAAAAAGAGTATTGACTTAAAGTCTAACCTATAGGATACTTACAGCCATCGAGAGGGACACGGCGAATAGCCATCCCAATCGACACCGGGGTCAACCGGATAAGTAGACAGcc                    <  1:283398/301‑1 (MQ=255)`  
                                                                                                                                                                                                                                                                                    `aTCTTAAAGTTTAAACATAAAGACCAGACCTAAAGACCAGACCTAAAGACACTACATAAAGACCAGACCTAAAGACGCCTTGTTGTTAGCCATAAAGTGATAACCTTTAATCATTGTCTTTATTAATACAACTCACTATAAGGAGAGACAACTTAAAGAGACTTAAAAGATTAATTTAAAATTTATCAAAAAGAGTATTGACTTAAAGTCTAACCTATAGGATACTTACAGCCATCGAGAGGGACACGGCGAATAGCCATCCCAATCGACACCGGGGTCAACCGGATACGTGGACAGCCtt                  >  1:108681/1‑300 (MQ=255)`  
                                                                                                                                                                                                                                                                                    `aTCTTAAAGTTTAAACATAAAGACCAGACCTAAAGACCAGACCTAAAGACACTACATAAAGACCAGACCTAAAGACGACTTGTTGTTAGCCATAAAGTGATAACCTTTAATCATTGTCTTTATTAATACAACTCACTATAAGGAGAGACAACTTAAAGAGACTTAAAAGATTAATTTAAACTTTATCAAAAAGAGTATTGACTTAAAGTCTAACCTATAGGATACTTACAGCCATCGAGAGGGACACGGCGAATAGCCATCCCAATCGACACCGGGGTCAACCGGATAAGTAGACAGCCTg                  >  1:51411/1‑301 (MQ=255)`  
                                                                                                                                                                                                                                                                                        `tAAAGGTTAAACATAAAGACCAGACCTAAAGACCAGACCTAAAGACACTACATAAAGACCAGACCTAAAGACGCCTTGTTGTTAGCCATAAAGTGATAACCTTTAATCATTGTCTTTATTAATACAACTCACTATAAGGAGAGACAACTTAAAGAGACTTAAAAGATTAATTTAAAATTTATCAAAAAGAGTATTGACTTAAAGTCTAACCTATAGGATACTTACAGCCATCGAGAGGGACACGGCGAATAGCCATCCCAATCGACACCGGGGTCAACCGGATAAGTAGACAGCCTGATaa              <  1:5476/301‑1 (MQ=255)`  
                                                                                                                                                                                                                                                                                        `tAAAGGTTAAACATAAAGACCAGACCTAAAGACCAGACCTAAAGACACTACATAAAGACCAGACCTAAAGACGCCTTGTTGTTAGCCATAAAGTGATAACCTTTAATCATTGTCTTTATTAATACAACTCACTATAAGGAGAGACAACTTAAAGAGACTTAAAAGATTAATTTAAAATTTATCAAAAAGAGTATTGACTTAAAGTCTAACCTATAGGATACTTACAGCCATCGAGAGGGACACGGCGAATAGCCAACCCAATCGACACCGTGGTCAACCGGATAAGTAGACAGCCTGATaa              <  1:79020/301‑1 (MQ=255)`  
                                                                                                                                                                                                                                                                                                   `cATAAAGACCAGACCTAAAGACCAGACCTAAAGACACTACATAAAGACCAGACCTAAAGACGCCTTGTTGTTAGCCATAAAGTGATAACCTTTAATCATTGTCTTTATTAATACAACTCACTATAAGGAGAGACAACTTAAAGAGACTTAAAAGATTAATTTAAAATTTATCAAAAAGAGTATTGACTTAAAGTCTAACCTATAGGATACTTACAGCCATCGAGAGGGACACGGCGAATAGCCATCCCAATCGACACCGGGGTCAACCGGATAAGTAGACAGCCTGATAAGTCGCACGaaa   >  1:15627/1‑301 (MQ=255)`  
                                                                                                                                                                                                                                                                                                   `cATAAAGACCAGACCTAAAGACCAGACCTAAAGACACTACATAAAGACCAGACCTAAAGACGCCTTGTTGTTAGCCATAAAGTGATAACCTTTAATCATTGTCTTAATTAATACAACTCACTATAAGGAGAGACAACTTAAAGAGACTTAAAAGATTAATTTAAAATTTATCAAAAAGAGTATTGACTTAAAGTCTAACCTATAGGATACTTACAGCCATCGAGAGGGACACGGCGAATAGCCATCCCAATCGACACCGGGGTCAACCGGATAAGTAGACAGCCTGATAAGTCGCACGaaa   >  1:147604/1‑301 (MQ=255)`  

                                                                                                                                                                                                                                                                                                    `|`                                                                                                                                                                                                                                                                                                              
`‑‑‑‑‑‑‑‑‑‑‑TCTCACAGTGTACGGACCTAAAGTTCCCCCATAGGGGGTACCTAAAGCCCAGCCAATCACCTAAAGTCAACCTTCGGTTGACCTTGAGGGTTCCCTAAGGGTTGGGGATGACCCTTGGGTTTGTCTTTGGGTGTTACCTTGAGTGTCTCTCTGTGT‑CCCTATCTGTTACAGTCTCCTAAAGTATCCTCCTAAAGTCACCTCCTAACGTCCATCCTAAAGCCAACACCTAAAGCCTACACCTAAAGACCCATCAAGTCAACGCCTATCTTAAAGTTTAAACATAAAGACCAGACCTAAAGACCAGACCTAAAGACACTACATAAAGACCAGACCTAAAGACGCCTTGTTGTTAGCCATAAAGTGATAACCTTTAATCATTGTCTTTATTAATACAACTCACTATAAGGAGAGACAACTTAAAGAGACTTAAAAGATTAATTTAAAATTTATCAAAAAGAGTATTGACTTAAAGTCTAACCTATAGGATACTTACAGCCATCGAGAGGGACACGGCGAATAGCCATCCCAATCGACACCGGGGTCAACCGGATAAGTAGACAGCCTGATAAGTCGCACGAAAA  >  NC_001604.1/1‑581`

  

Alignment Legend

Aligned base mismatch/match (shaded by quality score): `ATCG/ATCG` `< 3 ≤` `ATCG/ATCG` `< 11 ≤` `ATCG/ATCG` `< 23 ≤` `ATCG/ATCG` `< 35 ≤` `ATCG/ATCG` `< 38 ≤` `ATCG/ATCG`

Unaligned base: `atcg`    Masked matching base: `atcg`    Alignment gap: `‑`    Deleted base: `‑`

Reads not counted as support for junction

read\_name Not counted due to insufficient overlap past the breakpoint.

read\_name Not counted due to not crossing MOB target site duplication.
